# The Effect of Therapeutic Exercise on Long-Standing Adductor-Related Groin Pain in Athletes: Modified Hölmich Protocol

**DOI:** 10.1155/2018/8146819

**Published:** 2018-03-12

**Authors:** Abbas Yousefzadeh, Azadeh Shadmehr, Gholam Reza Olyaei, Nasrin Naseri, Zahra Khazaeipour

**Affiliations:** ^1^Physiotherapy Department, School of Rehabilitation, Tehran University of Medical Sciences, Tehran, Iran; ^2^Brain and Spinal Cord Injury Research Center, Neuroscience Institute, Tehran University of Medical Sciences, Tehran, Iran

## Abstract

**Objective:**

The Hölmich protocol in therapeutic exercise is the most appropriate method for the treatment of long-standing adductor-related groin pain (LSAGP). Herein, we evaluated a modified Hölmich protocol to resolve the possible limitations intrinsic to the Hölmich protocol in terms of the rate of return to sport and the recovery period for athletes with LSAGP.

**Design:**

The study followed a single-blind, before/after study design, where 15 athletes with LSAGP (mean age = 26.13 years; SD = 4.48) performed a 10-week modified Hölmich therapeutic exercise protocol.

**Results:**

Outcome scores related to pain, hip adductor and abductor muscle strengths, and the ratio of maximum isometric and eccentric hip adduction to abduction strength increased significantly. Likewise, hip abduction and internal rotation ROM improved significantly compared to that at baseline. Furthermore, functional records (*t*-test, Edgren Side Step Test, and Triple Hop Test) showed significant improvement after treatment. Finally, 13 athletes (86.6% of the participants) successfully returned to sports activity in a mean time of 12.06 weeks (SD = 3.41).

**Conclusion:**

The findings of this study objectively show that the modified Hölmich protocol may be safer and more effective than the Hölmich protocol in athletes with LSAGP in promoting their return to sports activity.* This trial is registered with*  IRCT2016080829269N1.

## 1. Introduction

Despite the fact that groin injury is prevalent among soccer players, representing 9%–16% of all soccer injuries [1, 2], this injury still frequently causes sport physiotherapists trouble due to difficulties in treatment. Groin injuries often present major problems, such as high rates of recurrence [3], prolonged durations of absence [1], unclear prognosis [4], and long-term symptoms [4, 5]. Reports indicate that 69% of groin injuries in soccer players are adductor-related [6, 7]. When the condition becomes long-standing, the athlete usually waits a relatively long period of time before returning to sports activity [7].

A review of the studies shows that therapeutic exercise is the most appropriate method for treating long-standing groin pain [4, 5, 7–9]. Hölmich et al. (1999) showed that therapeutic exercise (concentrated on hip and abdominal muscle strengthening) compared with physiotherapy including passive agents (stretching, TENS, transverse friction massage, and laser therapy) leads to better results in terms of reducing pain and returning to sports activity [4, 5, 10]. In the study by Hölmich et al. (1999), the average time from the start of treatment to the return to sports activity in the group treated with therapeutic exercise was 18.5 weeks. It seems that this recovery period (18.5 weeks) is too long because, in professional sports, there is often a lot of pressure to get an athlete back to his/her sport as soon as possible [5, 8, 11].

Since 1999, many studies have been conducted in the field of musculoskeletal lesion rehabilitation. These results may be useful for resolving the possible limitations of the Hölmich et al. protocol (regarding the group treated with therapeutic exercise) and, as a result, lead to a shortened period of recovery for athletes with LSAGP [11–15]. The potential limitation of the Hölmich protocol and related suggestions are described as follows:

1. Athletes treated with therapeutic exercise were not allowed to stretch the adductor muscles. Since stretching is a standard technique prescribed to realign the collagen fibers during muscle repair [12], we suggest that stretching should be a component of the exercise protocol.
2. In Module 2, Exercise 3 of the Hölmich et al. protocol [5], they used weight-pulling abduction/adduction, although it has been claimed that motor unit recruitment when using elastic bands is greater than when a weight machine or free weights are used [11]. On the other hand, hip adductor strengthening exercises using elastic bands have been introduced as dynamic, high intensity exercises and are some of the best exercises to be included in preventative and treatment plans for groin injuries [13]. Furthermore, elastic bands can be used anywhere and in any condition [13]. Because of the previous claims, we suggest using an elastic band as an external load for hip abduction/adduction exercises in the exercise protocol.
3. Changes in trunk muscle function [14] and weakness of core muscles have been suggested to be factors related to groin injuries in athletes [15, 16]. We suggest that there should be more emphasis on core exercises in the exercise program.
4. Sliding hip abduction/adduction exercises, used in Module 1 of the Hölmich et al. protocol [5], were painful for some participants in our pilot study and caused some side effects, so we chose not to use these exercises in the treatment program.
5. Athletes treated with therapeutic exercise after the 6th week of the treatment had permission to jog as long as it did not arouse groin pain. We believe that all participants should have an identical running program that teaches them how to progressively increase running speed, duration, and other parameters needed for returning to sports.

The Copenhagen Adduction (CA), a high-intensity exercise carried out to the hip's outer range of motion [13, 17], can be performed at the end stages of the treatment. CA strengthens both the hip adductors and abductors, preparing for muscular stability in the groin area [17]. Serner et al. [13] described the details of CA. The role of the physiotherapist during treatment should not only be one of supervision and instruction but also focus on increasing the intensity or resistance of the exercises at each successive weekend.

In the current study, according to the above-mentioned points, we developed a modified version of the Hölmich et al. protocol [5] for the treatment of LSAGP and aimed to evaluate its effects on athletes with this type of injury. The dependent variables in our study were pain, hip adduction/abduction muscle strength, hip abduction and internal rotation range of motion (ROM), functional ability, and mean time required to return to the sports activity.

## 2. Method

### 2.1. Subjects

Athletes were called via declaration in sport clubs. Sport physiotherapists and physicians were also asked to refer athletes with LSAGP to the physiotherapy clinic. After interviewing and examining 27 athletes, 18 of them, who each signed an informed consent form, were included in the study. The Ethics Committee of Tehran University of Medical Sciences approved the study. This study was conducted in accordance with the Declaration of Helsinki (1964). The included subjects were men aged 18–35 years (mean = 26.13 years; SD = 4.48) with a history of groin pain for at least two months (mean = 22.53 months; SD = 21.08); they had to be motivated to return to their prior level of sports activity; they had to have painful palpation of the adductor tendons and/or their attachment to the pubic bone and, finally, had to have groin pain of less than 6 (in our pilot study, the patients who had a pain score of 6 or more than 6 on the VAS during legs adduction against resistance could not perform functional tests; therefore, we considered this level of pain as the highest level to participate in the study) on adduction of legs against resistance, according to a visual analogue scale (VAS). Hölmich et al. [18] showed that the test used to evaluate groin pain is reliable. Moreover, at least two of the subsequent criteria had to be found: an obvious history of morning groin pain and stiffness, groin pain due to sneezing or coughing, night groin pain, pain at the symphysis pubis joint when palpated, or radiological signs suggestive of osteitis pubis.

The exclusion criteria were inguinal or femoral hernia, prostatitis or persistent urinary tract disease, backache felt from the T10 to L5 segments, pelvic or lower extremity fracture, or any other problems of the lower limbs prohibiting the subject from fulfilling the treatment course, with clinical findings demonstrating genitofemoral or ilioinguinal nerve entrapment, nonsteroidal anti-inflammatory drug consumption during the treatment course, osteoarthrosis or other disorders of the hip, or any other problem prohibiting the subject from fulfilling the treatment plan [5, 7]. Furthermore, athletes who had performed normative hip adductor strengthening exercises more than once a week in the 6 months prior to the study were excluded [19].

### 2.2. Design

The clinical trial was single-blind and was designed as a before/after study. The physiotherapist who provided the ratings was not involved in the treatment and was not informed of the treatment protocol.

### 2.3. Treatment

**Figure 1:**
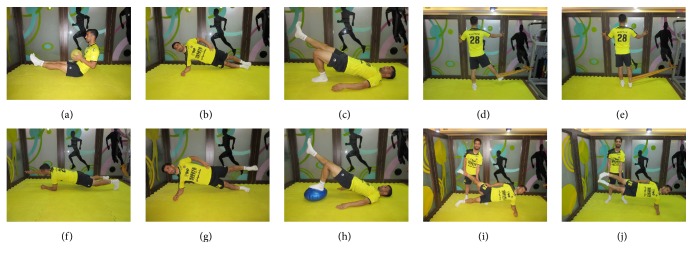
Some of the exercises that the participants performed during the first and second phases of modified Hölmich et al. protocol. Descriptions are provided in Tables 1 and 2.

We have provided the components of the modified Hölmich et al. protocol in Tables 1 and 2. Some of the exercises that are described in Tables 1 and 2 are shown in Figure 1. An experienced sport physiotherapist oversaw the strict implementation of this treatment protocol. We gave no therapy other than the therapeutic exercise and we did not allow any athletic activity during the treatment. The minimum duration of treatment was 10 weeks; however, the athletes could continue their treatment for up to 12 weeks if necessary. During the first two weeks, the participants performed part 1 of our protocol three times a week. From the third week on, they performed part 2 of the protocol three times a week (on even or odd days) and carried out the exercises from part 1 every other day. The duration of each session was approximately 120–150 min.

**Figure 2:**
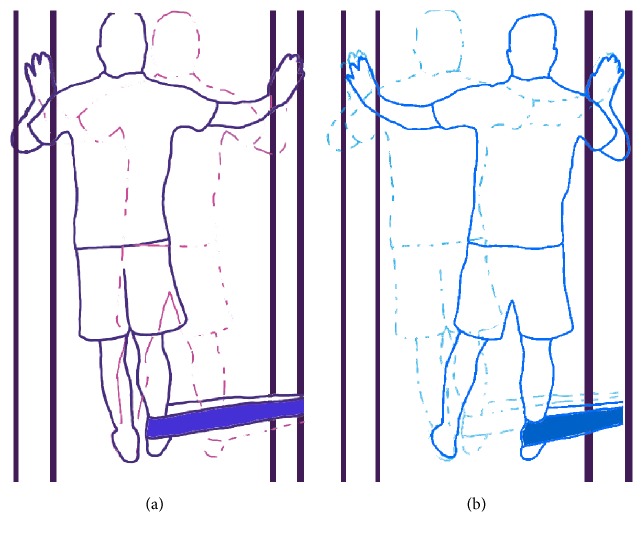
Isometric hip adduction. The subject tries to abstain from concentric or eccentric adductors contraction by moving his body in harmony with hip adduction (a) and coming back to the reference position (b).

In the first phase (part 1, Exercise 4), we had participants do isometric hip adduction using elastic bands (Thera-Band®, Akron, Ohio, USA). The subject moved his body in harmony with adduction and coming back to the reference position in order to prevent concentric and eccentric adductors contractions as much as possible (Figure 2). The time under tension for the isometric adduction was 10 sec [20].

In the second phase of the treatment, the participants performed hip adduction-abduction exercises using elastic bands in three consecutive phases of concentric, isometric, and eccentric contractions, as Jensen et al. [19] showed in their study (part 2, Exercise 4). The physiotherapist determined the resistance of the elastic band at the beginning of the treatment, which was the maximum resistance that could be performed by the athlete, pain-free, for 10 repetitions. The participants increased this load every treatment week under the supervision of the physiotherapist. In addition to our therapeutic exercise protocol, we allowed the participants to ride a bicycle during the first 6 weeks and, from the 6th week on, their running programs were started according to Hogan's return to running program [7, 20].

After the final assessment at the 10th week, we gave a similar written document, outlining the unique rehabilitation plan, to each athlete. We also conducted weekly telephone follow-up calls with each athlete to determine whether they had gone back to sports activities or not. The final follow-up assignment for the athletes at 20 weeks after the start of the treatment was to fill out a new questionnaire regarding their symptoms.

### 2.4. Outcome Measurements

At baseline and 10 weeks after that, a trained, single-blind physiotherapist evaluated the athletes. We did not allow the athletes to take part in any kind of competition or training the day prior to the first evaluation.

#### 2.4.1. Hip Muscle Strength (Adductor/Abductor)

We used a hand-held dynamometer (HHD) (Powertrack II Commander JTECH Medical, Salt Lake City, Utah, USA) for muscle strength measurement, which was previously reported to be valid [19, 21]. Maximal isometric hip abduction (IHAB), maximal isometric hip adduction (IHAD), maximal eccentric hip abduction (EHAB), maximal eccentric hip adduction (EHAD), and maximal IHAD/IHAB and EHAD/EHAB ratios were included in our primary outcome measurements for hip muscle strength. We also calculated the percentage gain in muscle strength due to the effect of the treatment. In this study, we defined the percentage gain as the difference between the before and after muscle strengths divided by the before strengths and multiplied by 100. We performed the measurements on the affected lower extremity.

The strength measurement procedures for hip adductors and abductors have been explained in detail in previous studies [20–22]. All the participants were recommended to a 10 min standardized warm-up program prior to the hip strength measurements. This warm-up program consisted of light running, squatting, and hip adduction and abduction muscle activation [20]. Using a make test in the supine position, we performed our measurements for IHAB and IHAD based on Thorborg et al. [21]. The athletes were asked to fix themselves by taking the sides of the examination table with their hands. The lower limb being tested was in a straight position and the knee and hip in the lower limb not being tested were in 90° flexion. The assessor exerted resistance in a fixed status 8 cm proximal to the most prominent point of the lateral malleol (for IHAB and medial malleol for IHAD) and the participant being tested performed an isometric maximum voluntary contraction (MVC) against the dynamometer and the assessor for 5 sec. The rest duration between each trial was 30 sec. The standardized command by the assessor was “go ahead-push-push-push-push and relax.” The individual test was performed four times and the average of the three highest amounts was reported. According to Thorborg et al. [20], using a break test with the athletes on their sides, we performed our measurements for EHAB and EHAD. The athletes were asked to fix themselves by taking the sides of the examination table with their hands. The lower limb being tested was in a straight position and the knee and hip in the lower limb not being tested were in 90° flexion. The assessor exerted resistance in a fixed status 8 cm proximal to the most prominent point of the lateral malleol (for EHAB and medial malleol for EHAD) and the participant being tested performed an isometric maximum voluntary contraction (MVC) against the dynamometer and the assessor for 3–5 sec before the break was carried out by the assessor. The rest duration between each trial was 60 sec. The standardized command by the assessor was “go ahead-push-push-push-push.” The single test was repeated till a force plateau of less than 5% between two sequential trials was attained and the average of these values was reported. The rest period between make and break tests was 5 min. Applying the lower limb length and body weight, we presented all force amounts as Newton-meters per kilogram of body weight (N·m·kg^−1^). We measured the leg length from the most prominent point of the anterior superior iliac spine to the most prominent point of the lateral malleol in supine position [21].

#### 2.4.2. Pain

We performed the pain assessment, based on the VAS, in two situations: (1) pain during the functional tests: the pain felt by the participants during each functional test was recorded and the average earned from the three functional tests was used for data analysis; (2) pain with adduction of legs against resistance: the participant was in supine position. The assessor physiotherapist stood at the end of the examination table with hands and forearms between the feet of the participant to hold them apart. The participant*ʼ*s feet were positioned upward and he pushed them together with maximal force without elevating the legs or pelvis. The pain felt by the participant was recorded for data analysis [3, 18].

#### 2.4.3. Functional Ability

We applied three functional tests including the* t*-test, Triple Hop Test for Distance (THT), and Edgren Side Step Test (ESST), as reliable and valid measures for the assessment of multiple agility components (unidirectional, bidirectional, and multidirectional motions), lower limb speed, and power [23–26]. All tests were conducted on a natural soccer pitch during the normal working day hours of 10 a.m. to 1 p.m. and the participants wore a soccer kit to reproduce the playing conditions. All participants became familiar with the testing method used in the current study before the actual test was applied. Immediately before testing, participants carried out a standard 25 min warm-up including 10 min of light running, 10 min of dynamic stretching, and 5 × 30 m of running exercises [27]. During testing, the air temperature ranged from 19°C to 26°C. Participants were asked to do each test twice and the best score obtained from two trials was recorded for data analysis. If the participant failed to fulfill a test in two trials due to disqualification, an extra trial was allowed. The rest duration between each trial was 60 sec and between each test was at least 3 min. Prior to testing, participants were given 1 to 3 (self-selected) practice trials [26].

**Figure 3:**
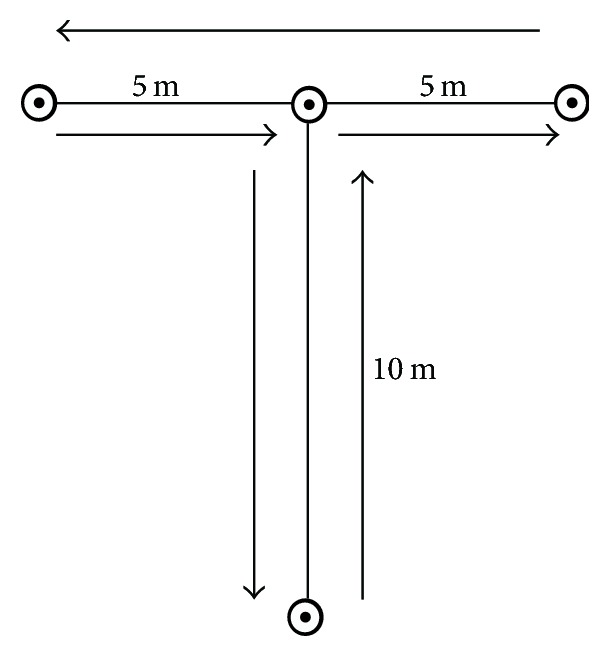
*t*-Test.

For the* t*-test, we designed three cones 5 m apart in a row and a fourth cone 10 m from the middle cone so that the cones form a T (Figure 3). The participants started at the base of the “T” (start line). On the command “go” by the rater, the participant ran to the middle cone as quickly as possible, then sidestepped 5 m to the right cone, next sidestepped 10 m to the far left cone, after that sidestepped 5 m back to the middle cone, and finally ran 10 m backwards as quickly as possible to pass the finish line. The rater began the chronometer on “go” and stopped when the participant passed the finish line. If the participant could not run the path as instructed, crossed his legs more than once during sidestepping, shook any cones, or could not keep his body and feet frontward during the test, he was disqualified and had to repeat the test [25, 26].

We measured the THT using a standard tape measure fixed to the ground perpendicular to a starting line. Each participant began the test by standing on the affected limb with the great toe on the starting line. The assessor physiotherapist measured the distance from the starting line to the point where the back of the participant*ʼ*s heel hit the ground upon completing three sequential hops on the affected limb. Disqualification was determined if the participant failed to perform a triple hop without losing balance and contacting the ground with the opposite foot [23, 24].

**Figure 4:**
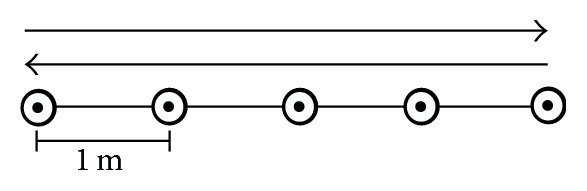
Edgren Side Step Test (ESST).

For the ESST, we placed five cones in a line, 1 m apart (Figure 4). The participant stood behind the far left cone and was asked not to cross his feet during sidestepping. On the “go” command, the participant sidestepped to the right till his right foot had contacted or passed the external cone and then sidestepped to the left till his left foot had contacted or passed the left external cone. The participant sidestepped backward and forward to the external cones as quickly as possible for 10 sec. The rater gave the participant one point per completion of each 1 m enhancement signed by a cone. If the far end cones were not attained, those points were not granted. Disqualification was determined if the participant failed to keep his body and feet frontward during the test, crossed his legs, or did not fulfill the course as instructed [26].

#### 2.4.4. Range of Motion (ROM) of the Hip Joint: Abduction and Internal Rotation

We measured the passive hip abduction ROM with the participant supine on the examination table. The starting position was with the legs in contact. The fixed arm of a goniometer was placed parallel to the line between the anterior superior iliac spines and the moving arm coincided with the longitudinal axis of the femur. The limb under test was passively abducted until rotation of the femur began, recording the end point of the measurement [22].

We measured passive internal rotation of the hip while the participant was in a prone position with knees at 90° flexion and ankles and knees both in contact. The participant was then asked to let the feet fall out bilaterally while keeping the knees at 90° flexion. To measure passive hip internal rotation, we aligned the moving arm of a standard goniometer with the long axis of the tibia, while the stationary arm was vertical [28]. We performed all ROM measurements once and on the affected lower limb.

### 2.5. Statistical Analysis

We maintained the statistician's blindness to the treatment protocol and outcomes until the analyses were complete. We used a double data entry process and SPSS Statistics, version 24, for the data analysis. We established the alpha level at .05 for all statistical analyses. The dependent variables had normal distribution (Kolmogorov-Smirnov test). To ascertain whether there were any considerable differences between the before and after amounts of the dependent variables, a paired-sample* t*-test was used.

## 3. Results

Eighteen athletes were initially included in the study, with 15 completing the treatment protocol. One athlete dropped out due to educational problems, one athlete withdrew because he had to go to military service, and one athlete was lost to follow-up. We have prepared basic characteristics of the study participants in Table 3.

Weekly follow-ups and a final follow-up at 20 weeks after the start of treatment showed that 13 athletes (86.6%) returned to full sports activity without groin symptoms in a mean time span of 12.06 weeks (SD = 3.41). Two athletes failed to return to their prior level of sporting activity and decided to change sports. We have explained the results of the measurements performed at the beginning and end of the treatment in the following subdivisions.

### 3.1. Hip Adductor and Abductor Muscle Strength

At the end of the treatment, the mean IHAD, IHAB, EHAD, and EHAB improved considerably compared to the baseline (Table 4). We also found considerable improvements in the ratio of mean, maximum, isometric and eccentric, and hip adduction to abduction strength, compared to the beginning of the treatment (Table 4). We have shown the percentage gain in muscle strength because of treatment in Table 4.

### 3.2. Visual Analogue Scale (VAS) for Pain

We found significant differences in VAS pain scores for the legs adduction against resistance. In addition, the VAS pain scores during the functional tests improved considerably compared to the scores at the start of the treatment (Table 4).

### 3.3. Functional Tests

THT and ESST measures increased considerably 10 weeks after treatment. Meaningful improvements were also found in the* t*-test agility scores. For details, refer to Table 4.

### 3.4. Hip Abduction and Adduction ROM

The ROM of the hip abduction and internal rotation increased significantly at the end of the treatment compared to the baseline. For details, refer to Table 4.

### 3.5. Adverse Effects

No adverse effects due to treatment were reported throughout the study.

## 4. Discussion

The aim of this study was to develop and evaluate a modified protocol based on exercise therapy for the treatment of LSAGP. Our findings suggest that this modified ten-week protocol that benefits from strengthening the muscles affecting the pelvis, core stabilization, hip adductor stretching, and high intensity eccentric exercise of the hip adductors may have a considerable effect on primary measured outcomes including pain, hip adductor and abductor muscle strength, hip ROM, functional ability, and returning to sport.

After completing our treatment protocol, 86.6% of the participants (*n* = 13) returned to their previous respective levels of sports activity, without groin symptoms. The mean time from baseline to completely pain-free sports activity was 12.06 weeks. These results were better than those obtained by Hölmich et al. [5] who reported a median time of 18.5 weeks for 79% of the participants in their therapeutic exercise regimen to return to their prior level of sports participation without groin pain. In our study, the higher rate of returning to full sports activity (86.6%) and shorter time for recovery (12.06 weeks) could be due to a variety of factors including use of a different method to strengthen hip adductor/abductor muscles (using elastic bands and emphasizing time under tension), use of core stabilization exercises, institution of high-intensity eccentric training for the adductors (Copenhagen Adduction), and hip adductor stretching. Furthermore, we tried to increase the level of difficulty of the exercises at every possible opportunity, by utilizing the expertise of a physiotherapist (part 1, Exercises 4 and 9; part 2, Exercises 4 and 10). We also applied the “return to running program” [29], whereas, in the Hölmich et al. (1999) protocol, there is no defined program for returning to running, and the subjects were ordered to begin running from the sixth week if it did not provoke groin pain. When there is no defined running program, the athlete may not be able to plan a graded return without causing further damage or he/she may be too cautious, due to fear of reinjury.

It should be mentioned that although the inclusion and exclusion criteria in the current study are almost similar to those of the study by Hölmich et al. [5], there are some differences between the two studies. The mean age of the participants in both studies is in a similar age group but the participants of the current study (mean age = 26.13) are a little younger than the participants of the study of Hölmich et al. [5] (mean age = 30). Furthermore, the athletes had to have pain less than 6 (based on VAS) on adduction of legs against resistance to be included in the current study, but there was no pain limit in the inclusion criteria of the study by Hölmich et al. [5]. In terms of the basic characteristics, there are also some differences between the two studies. For example, mean duration of injury in the participants of the current study (22.53 months) was much longer than those of the study by Hölmich et al. [5] (38 weeks). In addition, most of the participants in the exercise therapy group (71%) of the study of Hölmich et al. [5] had ceased their sports activities prior to the study, while in the current study, most of the participants (80%) had just reduced their sports activities at the baseline. These differences in inclusion and exclusion criteria and also basic characteristics may influence the results; therefore, we should be cautious in making our conclusion.

A higher percentage of participants, in a shorter period of recovery, could return to full sports participation in the current study as compared to the study by Weir et al. [7]. In their study, 55% of the athletes in the exercise therapy group could return to full sports activity after a median time of 17.3 weeks.

Furthermore, VAS pain scores changed considerably in the current study (from 5.07 to 0.27 in the legs adduction against resistance and from 5.20 to 0.73 during the functional tests) and were acquired in the relatively short duration of 10 weeks. These findings were better than those obtained by Weir et al. [7]. In the study by Weir et al. [7], VAS pain scores during sports participation were reduced from 58.5 at baseline to 21.0 (VAS of 100 equated to the highest level of pain), requiring sixteen weeks for these changes to occur, in the exercise therapy (ET) group.

The major differences that exist between the modified protocol applied in the current study and the program used by Hölmich et al. [5] which was subsequently reproduced by Weir et al. [7] in their ET group could explain the differences in results. Furthermore, the differences in results could be due to differences in supervision. The participants in the study by Weir et al. [7] were instructed by a physiotherapist as to how to perform the exercises on three separate occasions. The method of performing the exercises was controlled in these sessions, but the participants were not supervised while performing the exercises during the entire treatment period.

There are some differences between the inclusion and exclusion criteria and also the basic characteristics of the current study and the study of Weir et al. [7]. For example, unlike the present study, there was no pain limit in the inclusion criteria of the study by Weir et al. [7]. In addition, most participants (72%) in the study by Weir et al. [7] had ceased their sports activities prior to the study, but in the present study, most participants (80%) had just reduced their sports activities at the baseline. On the other hand, duration of injury in the participants of the present study (22.53 months) was much longer than that of the study by Weir et al. [7] (32 weeks). As we mentioned before, these differences may influence the results; therefore we should be cautious with our conclusion.

Hip joint abduction ROM in the affected limb improved significantly by the end of treatment (*P* = 0.0001). These results were similar to those obtained by Hölmich et al. [5]. They suggested that increased muscle strength, coordination, and reduced groin pain can lead to improved ROM. They also declared that adductor stretching might provoke the injury by causing pulling at the teno-osseous junction. The results of the current study are not consistent with these suggestions because all of the primary outcomes in our study improved significantly, despite adductor stretching being practiced by our participants. Furthermore, we did not observe any adverse effect in the current study. Notable point is that, in contrast to the present study, most participants in the study by Hölmich et al. [5] had stopped their sports activities prior to the study. The overall flexibility of the participants at the study baseline may have affected the results. More clinical trials are necessary to study the contribution of stretching in the treatment of LSAGP. Taylor et al. [28] declared that a decreased range of internal hip rotation might be a potential risk factor for groin injury [28]. Our findings showed a considerable increase in range of internal hip rotation after the treatment (*P* = 0.006). More clinical trials are needed to further support these results.

We found considerable improvements in IHAD and IHAB strength in the affected limb (by 58.79% (*P* = 0.0001) and 29.53% (*P* = 0.0001), resp.). EHAD and EHAB strength also increased considerably in the affected limb (by 54.66% (*P* = 0.0001) and 25.97% (*P* = 0.0001), resp.). As we did not have a control group in our study, it may be useful to compare these results with those obtained by similar studies performed in the future. Likewise, measurements related to functional ability improved considerably in the current study (*P* = 0.0001). Although we do not have any information about the participants' preinjury functional scores, it might be useful to compare our findings with those of identical future studies.

For the ratio of isometric and eccentric adduction strength to abduction strength, our results indicated a significant improvement after treatment (*P* = 0.006 and *P* = 0.009, resp.). It has been shown that an athlete with an eccentric adductor to abductor strength ratio of less than 80% is 17 times more likely to suffer from an adductor strain [22]. The ratio of eccentric adductor to abductor strength in the current study increased from 67% to 81% (refer to Table 4). These results can give us confidence that our athletes passed the high-risk zone, suggested by Tyler et al. [22], when the ratio is below 80%. Since there is no control group in the current study, the results related to ratio of adductor to abductor strength might be helpful for use in future studies.

The limitations of the current study are the small number of subjects and the lack of a control group. The number of initial participants in the present study (18 athletes) was too low to allocate half of them to the control group. Furthermore, the mean duration of symptoms in the participants was very long (22.53 months) and they had received many various treatments prior to their participation in the study; therefore it does not seem that our findings were the result of the time or placebo. However, the present study was strictly under supervision for strict implementation of the treatment protocol, blindness, and prevention of any therapy other than therapeutic exercise.

## 5. Conclusion

Although the current study was a small trial (*n* = 15) without controls, compared to the study by Hölmich et al. [5] (*n* = 29 in active training group), the findings of this single-blind, before and after clinical trial objectively show that therapeutic exercise based on our modified protocol may be safer and may also be more effective than the Hölmich et al. [5] therapeutic exercise protocol for LSAGP in athletes.

The outcome measures related to the ratio of eccentric adductor to abductor strength show that strengthening exercises should not be stopped after the treatment period. The athletes should be encouraged to continue the exercises, according to the given program, at the end of treatment. Future randomized clinical trials, with large sample sizes, should be very useful for evaluating the efficacy of this modified protocol.

## Figures and Tables

**Table 1:** Modified Hölmich et al. protocol: part 1.

Exercise	Amount	Rest period
(1) Brief warm-up using a stationary bicycle	10 min (25 W load at 20 km/h)	—

(2) Isometric, pain-free adduction against a soccer ball placed between the knees in the crook lying position	3 sets of 10 reps. (each rep. for 10 sec)	10 sec rest after each rep. and 2 min rest after each set

(3) Bilateral straight leg raising in a seated V position, as in Figure 1(a)	3 sets of 10 reps. (each rep. for 10 sec)	10 sec rest after each rep. and 2 min rest after each set

(4) Isometric standing hip adduction using elastic bands (both legs should be trained)	5 sets of 10 reps. (each rep. for 10 sec)	10 sec rest after each rep. and 2 min rest after each set

(5) Abdominal sit-ups in both straight and oblique directions	4 sets of 15 reps.	1 min rest after 15 consecutive reps.

(6) Prone bridging on forearms and toes (the back should be completely straight)	10 reps. (each rep. for 15 sec)	15–20 sec rest after each rep.

(7) Side bridging on the elbow (the trunk should be in neutral alignment, as in Figure 1(b))	10 reps. for each side (each rep. for 15 sec)	15–20 sec rest after each rep.

(8) Unilateral bridge exercise starting from the crook lying position (with one knee flexed and the opposite hip and knee extended so that trunk is in neutral spine alignment, as in Figure 1(c))	10 reps. of 12 sec (each rep. consists of 6 sec right leg raising followed by 6 sec of left leg raising)	15–20 sec rest after each rep.

(9) Wobble board balance training (beginning with both feet and gradual increase in difficulty by single leg standing and then adding some maneuvers like small knee bends to challenge the balance	8 min (the legs are switched once a minute when single leg standing)	—

**Table 2:** Modified Hölmich et al. protocol: part 2 (from the 3rd week onward).

Exercise	Amount	Rest period
(1) Brief warm-up using stationary bicycle	10 min (25 W load at 20 km/h)	—

(2) Low back extension exercise in the prone position with arms at the sides	3 sets of 10 reps.	30 sec rest after each set

(3) Abdominal sit-ups, in both straight and oblique directions, while holding a 3 kg medicine ball in hands	4 sets of 15 reps.	1 min rest after 15 consecutive reps.

(4) Standing hip add-abd. exercise with elastic bands (both legs) (Jensen et al., 2014). Figures 1(d) and 1(e) show the starting and end position of hip add. exercise	5 sets of 10 reps. for add. and 5 sets of 10 reps. for abd. (concentric phase = 1 sec and eccentric phase = 3 sec)	2–5 sec rest after each rep. and 1 min rest after each set

(5) Folding knife sit-ups beginning from the crook lying position, with a soccer ball located between the knees, simultaneous ab. sit-ups and hip flex (Hölmich et al., 1999)	5 sets of 10 reps.	1 min rest after 10 consecutive reps.

(6) In the prone position with arms stretched overhead, partial lifting of opposite arm and leg for 6 sec and then reverse sides	2 sets of 10 reps. (each rep. consists of 6 sec lift for one side and 6 sec for the opposite side)	6 sec rest after each rep. and 2 min rest after each set

(7) Prone bridging on forearms and toes with single limb lifting (Rt. arm, Lt. arm, Rt. leg, and Lt. leg are lifted consecutively, as in Figure 1(f))	8 reps. (6 sec for lifting each limb and total time of 24 sec for each rep.)	30 sec rest after each rep.

(8) Side bridging on the elbow plus single hip abd., as in Figure 1(g)	10 reps. for each side (each rep. for 10 sec)	15–20 sec rest after each rep.

(9) Unilateral bridge exercise. Start from crook lying position with one knee flexed and the opposite hip and knee extended while the flexed limb is on an unstable surface such as a Dyna Disc®, as in Figure 1(h)	10 reps. of 12 sec (each rep. consists of 6 sec Rt. leg raising followed by 6 sec Lt. leg raising)	15–20 sec rest after each rep.

(10) Wobble board balance training including small knee bends, catching and throwing a ball, hands touching the standing foot alternately, and gentle side kicking of the ball (during end weeks)	10 min (the legs are alternated)	

(11) Single leg, cross-country skiing (Hölmich et al., 1999)	5 sets of 10 reps. for each leg	1 min rest after 1 set for each leg

(12) Copenhagen Adduction exercise beginning from the 7th week, if it does not provoke pain (Ishøi et al., 2016; Serner et al., 2014), as in Figures 1(i) and 1(j)	Begin with 2 sets of 6 reps., can progress to 3 sets of 6 reps. and then to 3 sets of 10 reps. when it does not provoke pain	3–5 min rest after each set

(13) Pain-free adductor stretching in a sitting position with flexed knees and feet together	5 reps. of 15 sec	

*Note*. add.: adduction; abd.: abduction; rep.: repetition; ab.: abdominal; Lt.: left; Rt.: right; sec: second.

**Table 3:** Baseline characteristics of the participants (*n* = 15).

Age (years)	26.13 (SD = 4.48)
Height (meters)	179.67 (SD = 3.47)
Sports	Soccer = 14; runner = 1
Preferred limb	Right = 11; left = 4
Location of injury	Right = 6; left = 9
Duration of injury (months)	22.53 (SD = 21.08)
Level of athletic activity	Elite (>5 times per week) = 4
	Subelite (3 or 4 times per week) = 11
Sports activity at baseline	Ceased = 2; reduced = 12; unchanged = 1
Pain (VAS^**∗**^) on legs adduction against resistance	5.07 (SD = 0.59)
Pain (VAS) during functional tests	5.20 (SD = 0.67)

^**∗**^VAS: visual analogue scale (no pain = 0; maximum pain = 10).

**Table 4:** Dependent variables, before and after values, and significance level after the paired samples *t*-test.

Dependent variable	Before	After	Percentage gain in muscle strength	Paired samples test sig. (two-tailed)
VAS pain score (legs add. against resistance)	5.07 (0.59)	0.27 (0.45)		0.0001
VAS pain score (during function)	5.20 (0.67)	0.73 (0.79)		0.0001
Hip Abd. ROM (°)	45.53 (4.10)	48.67 (4.25)		0.0001
Hip Int. rot. ROM (°)	23.40 (8.73)	25.13 (8.21)		0.006
Iso. Add. (N·m·kg^−1^)	1.26 (0.49)	2.00 (0.46)	58.79%	0.0001
Iso. Abd. (N·m·kg^−1^)	1.63 (0.33)	2.12 (0.31)	29.53%	0.0001
Ecc. Add. (N·m·kg^−1^)	1.61 (0.75)	2.49 (0.66)	54.66%	0.0001
Ecc. Abd. (N·m·kg^−1^)	2.44 (0.54)	3.07 (0.66)	25.97%	0.0001
Ratio of Iso. hip Add. to Abd.	0.77 (0.24)	0.94 (0.15)		0.006
Ratio of Ecc. hip Add. to Abd.	0.67 (0.27)	0.81 (0.16)		0.009
*t*-Test (sec)	11.51 (0.91)	9.86 (0.51)		0.0001
ESST (m)	23.80 (2.78)	29.80 (2.42)		0.0001
THT (m)	5.30 (0.56)	6.29 (0.39)		0.0001

*Note*. Abd.: abduction; Add.: adduction; Int. rot.: internal rotation; Iso.: isometric; Ecc.: eccentric; VAS: visual analogue scale; ESST: Edgren Side Step Test; THT: Triple Hop Test for Distance.
